# Lifetime and past-month substance use and injection among street-based female sex workers in Iran

**DOI:** 10.1186/s12954-021-00477-5

**Published:** 2021-03-16

**Authors:** Payam Roshanfekr, Mehrdad Khezri, Salah Eddin Karimi, Meroe Vameghi, Delaram Ali, Sina Ahmadi, Elahe Ahounbar, Kambiz Mahzari, Mohsen Roshanpajouh, Mehdi Noroozi, Mostafa Shokoohi, Ali Mirzazadeh

**Affiliations:** 1 Social Welfare Management Research Center, University of Social Welfare and Rehabilitation Sciences, Tehran, Iran; 2 HIV/STI Surveillance Research Center, and WHO Collaborating Center for HIV Surveillance, Institute for Futures Studies in Health, Kerman University of Medical Sciences, Kerman, Iran; 3 Social Determinants of Health Research Center, Tabriz University of Medical Sciences, Tabriz, Iran; 4 Social Determinants of Health Research Center, University of Social Welfare and Rehabilitation Sciences, Tehran, Iran; 5 Substance Abuse and Dependence Research Center, University of Social Welfare and Rehabilitation Sciences, Tehran, Iran; 6 Advisor to the Committee on Aids Prevention and Control Affiliated to the Health Ministry, Expert, Prevention and Addiction Affairs Bureau State Welfare Organization (SWO), Tehran, Iran; 7 Addiction Department, School of Behavioral Sciences and Mental Health (Tehran Institute of Psychiatry), Iran University of Medical Sciences (IUMS), Tehran, Iran; 8 Dalla Lana School of Public Health, University of Toronto, Toronto, ON Canada; 9 Department of Epidemiology and Biostatistics, University of California San Francisco, San Francisco, CA USA

**Keywords:** Female sex workers, Drug use, Injection drug use, Harm reduction, Iran

## Abstract

**Background:**

Street-based female sex workers (FSWs) are highly at risk of HIV and other harms associated with sex work. We assessed the prevalence of non-injection and injection drug use and their associated factors among street-based FSWs in Iran.

**Methods:**

We recruited 898 FSWs from 414 venues across 19 major cities in Iran between October 2016 and March 2017. Correlates of lifetime and past-month non-injection and injection drug use were assessed through multivariable logistic regression models. Adjusted odds ratios (AOR) and 95% confidence intervals (CI) were reported.

**Results:**

Lifetime and past-month non-injection drug use were reported by 60.3% (95% CI 51, 84) and 47.2% (95% CI 38, 67) of FSWs, respectively. The prevalence of lifetime and past-month injection drug use were 8.6% (95% CI 6.9, 10.7) and 3.7% (95% CI 2.6, 5.2), respectively. Recent non-injection drug use was associated with divorced marital status (AOR 2.00, 95% CI 1.07, 3.74), temporary marriage (AOR 4.31 [1.79, 10.40]), had > 30 clients per month (AOR 2.76 [1.29, 5.90]), ever alcohol use (AOR 3.03 [1.92, 6.79]), and history of incarceration (AOR 7.65 [3.89, 15.30]). Similarly, lifetime injection drug use was associated with ever alcohol use (AOR 2.74 [1.20–6.20]), ever incarceration (AOR 5.06 [2.48–10.28]), and ever group sex (AOR 2.44 [1.21–4.92]).

**Conclusions:**

Non-injection and injection drug use are prevalent among street-based FSWs in Iran. Further prevention programs are needed to address and reduce harms associated with drug use among this vulnerable population in Iran.

## Introduction

The high prevalence of drug use among female sex workers (FSWs) has been reported in different international studies [1, 2]. A reason offered for the high prevalence drug use among FSWs is a coping strategy for facilitating the process of engaging sexually with clients [3]. Literature has also documented the role of drug use in promoting HIV risk among FSWs [4]. In comparison with women in the general population, FSWs are more likely to become HIV-infected. For example, a meta-analysis estimated that FSWs are 13.5 times more likely to be living with HIV than other women of similar reproductive age in low- and middle-income countries [5]. Drug use, large number of paid and nonpaid sexual partners, condomless sexual intercourse, and involvement in group sex practices are of some possible explanations for the increased risk of HIV infection among FSWs [6, 7].

The evidence has shown that street-based FSWs experience multiple vulnerabilities, including police harassments, sexual violence, large number of clients, elevated risk of unprotected sex, and higher HIV/STI prevalence greater than another type of FSWs [8–11]. Studies also showed that street-based FSWs are more likely to report drug use than non-street-based FSWs [12, 13]. A study reported that about 60% of street-based FSWs in Addis Ababa abused substances [14]. Another study in US reported that their participants were mostly poly drug users, and 80.4% and 68.2% of street-based FSWs reported alcohol use and crack-cocaine use, respectively [15].

In Iran, FSWs have been recognized as the second most at-risk subpopulation for HIV infection. Population size estimation study estimated that 228,700 women were engaged in sex work in 2017 [16]. Studies using data from two national surveillance surveys showed that HIV prevalence among FSWs was 4.5% in 2010 [17] and 2.1% in 2015 [18], and a recent meta-analysis reported the pooled HIV prevalence as 2.23% among FSWs in Iran [19]. While our understanding about non-injection and injection drug use among FSWs is growing in Iran [20], less attention has been paid to the pattern of injection and non-injection drug use specifically among street-based FSWs. Studies with facility-based participants have estimated that that 24.9% and 14.6% of FSWs reported past-month non-injection drug use [21] and lifetime injection drug use [22]. Crystal methamphetamine use also has been reported by 15.0% of FSWs in Iran [23].

Similar to other countries, there are different FSWs types in Iran. In Iran, while the majority operate sex work in small groups or as an individual-based business, some of them are linked to some homes, recognized as “illegal brothels” where they can meet clients. Others seek clients in public venues, including parks, shopping centers, and street corners. The typology of sex workers, their social networks, and physical presence have been overlooked in investigations; however, experts believe the street-based FSWs form the most high-risk subgroups, have multiple risk profiles, and need to be targeted first by prevention program [24]. The present study approached and recruited street-based FSWs from public venues for the first time. We applied a rapid assessment and response method to measure the prevalence rate of non-injection and injection drug use and factors associated with such behaviors.

## Methods

### Study design

This cross-sectional survey was performed to measure the high-risk behaviors of street-based FSWs in Iran using a rapid assessment and response method. We recruited 898 FSWs from 414 venues across 19 major cities in Iran between October 2016 to March 2017. Venues consisted of public places where FSWs mostly attend for socializing, seeking clients, or drugs. The selection of the venues was carried out by the feedback of the key informants and local experts. The venues also were chosen based on their population size and locations after our team visited all reported places. The random sampling method was used from the universe of venues to recruit FSWs.

A convenience sample of 3–5 eligible participants were recruited at each venue. Inclusion criteria were: being ≥ 18 years old, have had sex for money or goods (food, drugs, etc.) in the past year, worked or lived in the city at the time of the study, and provided verbal consent were invited to participate in the study.

### Data collection

Trained female interviewers have met with FSWs participants individually and in private and asked the questions using the questionnaire and recorded the relevant responses. The questionnaire was previously piloted and used in two national bio-behavioral surveillance surveys among FSWs in Iran [18, 25]. The survey questionnaire was in Farsi and included various sections such as demographics, history of non-injection and injection drug use, sexual practices, and access to prevention and care services.

### Measures

The two primary study outcomes for this paper were lifetime and past-month drug use and injection frequencies. They both have been explored by self-report measures. We considered any self-reported history of drug use (which also includes injection) in the past as lifetime drug use (binary variable: yes/no), and any self-reported history of drug injection as lifetime drug injection (binary variable: yes/no). Using the same approach, we also measured and defined non-injection and injection drug use in the past month.

We explored correlates of non-injection and injection drug use with independent variables of interest included age at interview (18–24, 25–34, or > 35 years), education level (never attended school, primary school, secondary school, high-school, diploma or college degree), marital status (single (never married), currently married, divorced, temporarily married, or widowed), age at first sex (≤ 18 or > 18 years), income ($230 or less, $230 to $1100, $1100 to $2200, or $2200 or more), age at sex work debut (≤ 18, > 18 years), the duration of sex work involvement (< 5 or ≥ 5), ever practiced group sex (yes or no), ever consumed alcohol (yes or no), and ever incarcerated (yes or no).

### Statistical analysis

We reported the prevalence of study outcomes by point and 95% Confidence Interval (CI). We initially examined the association between other variables and study outcomes in bivariate analysis using the Chi-squared test (or Fisher’s exact test). Those variables with *P* < 0.2 were included in a multiple logistic regression model. Based on the literature, we included all known confounders, such as education and marital status in the model, even if the crude analysis p value was not < 0.2. We reported the Adjusted Odds Ratio (AOR) point estimate and 95% CI as the effect measures. We used Stata version 11 (Stata Corp.) for data analysis. Moreover, *P* < 0.05 were considered as statistically significant.

### Ethical considerations

Given the criminalized nature of sex work in Iran and the low levels of literacy among a significant subset of the participants, which posed challenges to written informed consent, the interviewed FSWs provided verbal informed consent. The study participants were also briefed about the voluntary nature of their participation, objectives of the survey, the provided incentives, and the anonymity of all collected data.

## Results

### Participant characteristics

A total of 898 FSWs were recruited from 414 VDTs in 19 major cities of Iran. The response rate was 93.1%. The mean (SD) age of participants was 33.07 (7.94) years. Most participants (39.4%) were divorced at the time of the study and had secondary school education (27.3%). 21.8% earned $2200 or more per month, and about one-third of respondents (25.9%) were classified as homeless. With respect to sexual practices, the majority (70.2%) of participants had first sexual contact < 18 years old, and 30.8% reported entering sex work before the age of 18. Ever alcohol use was reported by 55.4%. Lifetime history of incarceration was reported by 28.7% (Table 1).

**Table 1:** Characteristics of street-based female sex workers in Iran by the subgroups of Injection and Non-injection drug use, 2017

	Non-injection drug use				Injection drug use				Total *N* (%)
	Never *N* (%)	Lifetime^a^ *N* (%)	Past-month *N* (%)	*P* value^b^	Never *N* (%)	Lifetime *N* (%)	Past-month *N* (%)	*P* value	
Overall	356 (39.64)	542 (60.36)	424 (47.22)		820 (91.31)	78 (8.69)	34 (3.78)		898 (100)
Age at interview									
18–24	58 (17.79)	59 (11.15)	50 (12.02)	0.003 (0.489)	111 (14.29)	6 (7.69)	3 (8.82)	0.069 (0.109)	117 (13.68)
25–34	149 (45.71)	225 (42.53)	172 (41.35)		344 (44.27)	30 (38.46)	17 (50)		374 (43.74)
≥ 35	119 (36.50)	245 (46.31)	194 (46.63)		322 (41.44)	42 (53.85)	14 (41.18)		364 (42.57)
Education									
Never attended school	25 (7.33)	37(6.86)	27(6.40)	0.042 (0.133)	57 (7.11)	5 (6.41)	2 (5.88)	0.035 (0.593)	62 (7.05)
Primary school	75 (21.99)	146 (27.09)	114 (27.01)		193 (24.06)	28 (35.90)	13 (38.24)		221 (25.11)
Secondary school	82 (24.05)	159 (29.50)	134 (31.75)		216 (26.93)	25 (32.05)	8 (23.53)		241 (27.39)
High school	71 (20.82)	84 (15.53)	61 (14.45)		149 (18.58)	6 (7.69)	4 (11.76)		155 (17.61)
Diploma	62 (18.18)	80 (14.84)	61 (14.45)		133 (16.58)	9 (11.54)	6 (17.65)		142 (16.14)
College	26 (7.62)	33 (6.12)	25 (55.92)		54 (6.73)	5 (6.41)	1(2.94)		59 (6.70)
Income									
$230 USD or less	27 (9.31)	77 (15.91)	61 (15.95)	< 0.001 (0.028)	93 (26.71)	11 (15.28)	4 (12.90)	0.017 (0.113)	104 (13.44)
$230 to $1100 USD	87 (30)	164 (33.88)	119 (31.15)		218 (31.05)	33 (45.83)	16 (51.61)		251 (32.43)
$1100 to $2200 USD	120 (41.38)	130 (26.86)	105 (27.49)		229 (32.62)	21 (29.17)	11 (35.48)		250 (32.30)
$2200 USD or more	56 (19.31)	113 (23.35)	97 (25.39)		162 (23.08)	7 (9.72)	0 (0)		169 (21.83)
Marital status									
Single (never married)	139 (39.04)	94 (17.34)	69 (16.27)	< 0.001 (0.000)	219 (26.70)	14 (17.95)	8 (23.53)	0.005 (0.684)	2333 (25.95)
Currently married	51 (14.33)	98 (18.08)	66 (15.57)		141 (17.20)	8 (10.26)	2 (5.88)		149 (16.59)
Divorced	115 (32.30)	239 (44.10)	207 (48.82)		323 (39.39)	31 (39.74)	12 (35.29)		354 (39.42)
Temporarily married^d^	18 (5.06)	66 (12.18)	48 (11.32)		73 (8.90)	11 (14.10)	6 (17.65)		84 (9.35)
Widowed	33 (9.27)	45 (8.30)	34 (8.02)		64 (7.80)	14 (17.95)	6 (17.65)		78 (8.69)
Housing status									
Living with family, friends, partner or spouse	208 (66.67)	284 (57.03)	211 (54.81)	< 0.001 (0.012)	458 (62.23)	34 (45.95)	15 (46.88)	0.008 (0.352)	492 (60.74)
Living in their own house	54 (17.31)	54 (10.84)	38 (9.87)		98 (13.32)	10 (13.52)	6 (18.75)		108 (13.33)
Homeless	50 (16.03)	160 (32.13)	136 (35.32)		180 (24.46)	30 (40.54)	11 (34.38)		210 (25.93)
Age at first sexual contact^e^									
≤ 18 versus > 18	180 (59.41)	395 (76.55)	318 (77.56)	< 0.001 (0.591)	514 (69.27)	61 (79.22)	23 (69.70)	0.069 (0.198)	575 (70.22)
Age at sex work initiation^f^									
≤ 18 versus > 18	82 (30.15)	142 (31.28)	101 (28.37)	0.750 (0.007)	199 (30.47)	25 (34.25)	11 (34.38)	0.508 (0.634)	224 (30.85)
Ever used alcohol									
Yes	180 (59.41)	378 (69.74)	295 (69.58)	< 0.001 (0.605)	435 (53.05)	63 (80.77)	25 (73.53)	< 0.001 (0.510)	498 (55.46)
No	120 (40.59)	164 (30.26)	129 (30.42)		385 (46.95)	15 (19.23)	9 (26.47)		400 (44.54)
Ever incarcerated									
Yes	34 (9.55)	224 (41.33)	167 (39.39)	< 0.001 (0.200)	210 (25.61)	48 (61.54)	13 (38.24)	< 0.001 (0.003)	258 (28.73)
No	322 (90.45)	313 (58.67)	257 (60.61)		610 (74.39)	30 (38.46)	21 (61.76)		640 (71.27)

^a^Lifetime also includes past-month drug use/injection

^b^Lifetime (past-month)

^c^Drug use also includes drug injection, and so the numbers in the subgroups do not add up to the total number

^d^Temporary marriage is locally named Sigheh

### Non-injection and injection drug use prevalence

Lifetime and past-month non-injection drug use was reported by 60.3% (95% CI 57.0, 63.5) and 47.2% (95% CI 43.9, 50.5) of participants. The prevalence of lifetime and past-month injection drug use were 8.6% (95% CI 6.9, 10.7) and 3.7% (95% CI 2.6, 5.2), respectively (Table 2).

**Table 2:** Prevalence of lifetime and past-month non-injection drug use and drug injection among street-based female sex workers in Iran, 2017

	Non-injection drug use				Injection drug use			
	% Lifetime	95% CI	% Past-month	95% CI	% Lifetime	95% CI	% Past-month	95% CI
Overall	60.36	57.07, 63.57	47.22	43.91, 50.54	8.69	6.93, 10.72	3.78	2.64, 5.25
Age at interview								
18–24	50.43	41.03, 59.79	42.73	33.63, 52.21	5.12	1.19, 1.08	2.56	0.53, 7.31
25–34	60.16	55.00, 65.15	45.99	40.85, 51.18	8.02	5.47, 11.25	4.54	2.66, 7.17
≥ 35	67.31	62.22, 72.10	53.30	48.02, 58.51	11.54	8.44, 15.27	3.85	2.11, 6.36
Education								
Never attended school	59.68	46.44, 71.94	43.55	30.99, 56.74	8.06	2.67, 17.82	3.22	0.39, 11.17
Primary school	66.06	59.41, 72.27	51.58	44.78, 58.33	12.67	8.58, 17.78	5.88	3.16, 9.84
Secondary school	65.97	59.61, 71.93	44.60	49.08, 61.97	10.37	6.82, 14.93	3.32	1.44, 6.43
High school	54.19	46.01, 62.21	39.35	31.61, 47.51	3.87	1.43, 8.23	2.58	0.70, 6.57
Diploma or college	56.22	49.06, 63.18	42.79	35.84, 49.93	69.96	38.59, 11.41	3.48	1.41, 7.04
Marital status								
Single (never married)	40.34	33.98, 46.94	29.61	23.83, 35.92	6.00	3.32, 9.87	3.43	1.49, 6.65
Currently married	65.77	57.56, 73.34	44.29	36.16, 52.65	5.36	2.34, 10.30	1.34	0.16, 4.76
Divorced	67.51	62.36, 72.36	58.47	53.14, 63.65	8.76	6.02, 12.19	3.39	1.76, 5.84
Temporarily married	78.57	68.26, 86.77	57.14	45.87, 67.89	13.09	6.72, 22.22	7.43	2.66, 14.90
Widowed	57.69	45.97, 68.80	43.59	32.38, 55.29	17.95	10.17, 28.27	7.69	2.87, 15.99
Income								
$230 USD or less	74.04	64.51, 82.14	58.65	48.5, 68.22	10.58	5.39, 18.13	3.85	1.05, 9.55
$230 to $1100 USD	63.34	59.09, 71.21	47.41	41.09, 53.78	13.15	9.22, 17.96	6.37	3.68, 10.14
$1100 to $2200 USD	52	45.61, 58.33	42	35.80, 48.38	8.4	5.27, 12.55	4.4	2.21, 7.73
$2200 USD or more	66.86	59.22, 73.90	57.40	49.57, 64.95	4.14	1.68, 8.34	0.00	0.00, 2.15
Housing status								
Living with family, friends, partner or spouse	57.72	53.22, 62.13	42.89	38.46, 47.39	6.91	4.83, 9.52	3.05	1.71, 4.97
Living in their own house	50	40.22, 59.77	35.18	26.24, 44.96	9.25	4.52, 16.36	5.55	2.06, 11.70
Homeless	76.19	69.84, 81.78	64.76	57.88, 71.21	14.28	9.85, 19.76	5.24	2.64, 9.17
Duration of sex work								
≤ 5	53.05	45.11, 60.87	42.07	34.41, 50.01	5.49	2.53–10.16	3.05	0.9, 6.97
> 5	66.85	62.68, 70.83	52.62	48.28, 56.92	11.80	9.18–14.84	5.06	3.35, 7.27
Age at first sex (years)								
≤ 18	68.69	64.72, 72.46	55.30	51.13, 59.41	10.60	8.21, 13.41	4	2.55, 5.94
> 18	49.59	43.15, 56.04	37.70	31.60, 44.11	6.56	3.79, 10.43	4.10	1.98, 7.40
Age at sex work debut (years)								
≤ 18	63.39	56.71, 69.70	45.09	38.45, 51.85	11.16	7.35, 16.03	4.91	2.47, 8.61
> 18	62.15	57.74, 66.41	50.80	46.33, 55.25	9.56	7.13, 12.47	4.18	2.60, 6.32
Number of clients per month								
< 5	55.40	48.45, 62.19	30.52	24.40, 37.17	6.57	3.63, 10.78	2.82	1.04, 6.03
6–15	56.22	48.74, 63.48	42.16	34.95, 49.62	12.43	8.04, 18.06	7.03	3.79, 11.17
16–30	71.75	63.22, 79.27	61.83	52.93, 70.17	12.98	7.74, 19.96	6.11	2.67, 11.67
> 30	82.5	74.50, 88.82	79.17	70.80, 86.04	7.5	3.48, 13.76	0.8	0.02, 4.5
Group sex (ever)								
No	60.24	56.13, 64.24	45.44	41.33, 49–58	7.23	5.25, 9.64	3.10	1.84, 4.85
Yes	60.75	58.81, 70.35	53.96	47.90, 59.92	12.23	8.62, 16.66	5.39	3.05, 8.74
Ever consumed alcohol								
No	41	36.13, 45.99	32.25	27.69, 37.07	3.75	2.11, 6.10	2.25	1.03, 4.22
Yes	75.90	71.89, 79.59	59.24	54.77, 63.58	12.65	9.85, 15.89	5.02	3.27, 7.32
Ever incarcerated								
No	49.69	45.74, 53.63	40.16	36.33, 44.07	4.69	3.18, 6.62	3.28	2.04, 4.97
Yes	86.82	82.07, 90.69	64.73	58.56, 70.55	18.60	14.04, 23.89	5.04	2.70, 8.46

### Non-injection drug use and associations with covariates

A higher prevalence of lifetime non-injection drug use was reported among FSWs who were in the older age category (46.3%), had secondary school level of education (29.5%), earned $230 to $1100 (33.8%), were divorced (44.1%), reported living with family, friends, partner or spouse (57.0%), had experienced first sex at a younger age (76.5%), ever consumed alcohol (69.7%), and had not ever incarcerated (58.6%). Moreover, non-injection drug use in the past month was significantly higher among participants who earned $230 to $1100 (31.1%), were divorced (48.8%), reported living with family, friends, partner or spouse (54.8%), and had initiated sex work at an older age (71.7%) (Table 1).

In the multivariable logistic regression model, lifetime non-injection drug use was more likely to be reported by FSWs who were divorced (AOR 2.00, 95% CI 1.07, 3.74) or temporarily married (AOR 4.31, 95% CI 1.79, 10.40), had > 30 clients per month (AOR 2.76, 95% CI 1.29, 5.90), ever consumed alcohol (AOR 3.03, 95% CI 1.92, 6.79), and had a history of incarceration (AOR 7.65, 95% CI 3.89, 15.30). Lifetime non-injection drug use was less likely to be reported by FSWs who had earned $230 to $1100 (AOR 0.34, 95% CI 0.14, 0.80), had experienced first sex at a younger age (AOR 0.47, 95% CI 0.29, 0.77). In addition, past-month non-injection drug use was also more likely to be reported by FSWs who were divorced (AOR 3.26, 95% CI 1.72, 6.16) or temporarily married (AOR 3.54, 95% CI 1.55, 8.08), had ˃30 clients per month (AOR 5.76, 95% CI 2.82, 11.72), ever consumed alcohol (AOR: 1.90, 95% CI 1.22, 2.97), and ever incarcerated (AOR 3.05, 95% CI 1.85, 5.01). Last-month non-injection drug use was less likely to be reported by FSWs who had experienced first sex at a younger age (AOR 0.45, 95% CI 0.26, 0.75) (Table 3).

**Table 3:** Correlates of injection and non-injection drug use among street-based female sex workers in Iran, 2017

Variable	Lifetime non-injection drug use			Past-month non-injection drug use			Lifetime injection drug use			Past-month injection drug use		
	AOR^a^	CI 95%	*P*	AOR	CI 95%	*P*	AOR	CI 95%	*P*	AOR	CI 95%	*P*
Age at interview												
18–24	Ref			Ref			Ref					
25–34	2.07	0.97, 4.39	0.058	0.88	0.41, 1.89	0.756	1.01	0.22, 4.57	0.993			
≥ 35	1.94	0.85, 4.42	0.114	0.85	0.36, 2.00	0.716	1.13	0.23, 5.54	0.877			
Marital status												
Single (never married)	Ref			Ref			Ref			Ref		
Currently married	1.90	0.89, 4.08	0.096	1.54	0.72, 3.30	0.264	0.11	0.01, 0.57	0.009	0.49	0.09, 2.48	0.393
Divorced	2.00	1.07, 3.74	0.028	3.26	1.72, 6.16	< 0.001	0.61	0.23, 1.56	0.304	0.58	0.20, 1.67	0.320
Temporarily married	4.31	1.79, 10.40	0.001	3.54	1.55, 8.08	0.003	1.76	0.56, 5.47	0.326	1.94	0.56, 6.66	0.292
Widowed	1.17	0.46, 2.95	0.731	1.62	0.65, 4.01	0.259	1.30	0.38, 4.43	0.675	1.31	0.35, 4.88	0.689
Income (per month)												
$230 USD or less	Ref			Ref			Ref					
$230 to $1100 USD	0.65	0.28, 1.50	0.313	0.57	0.25, 1.28	0.172	0.72	0.22, 2.32	0.588			
$1100 to $2200 USD	0.34	0.14, 0.80	0.014	0.60	0.26, 1.35	0.219	0.49	0.14, 1.62	0.242			
$2200 USD or more	0.49	0.19, 1.25	0.137	0.67	0.27, 1.63	0.381	0.22	0.05, 0.91	0.037			
Housing status												
Living with family, friends, partner or spouse	Ref			Ref			Ref			Ref		
Living in their own house	1.02	0.51, 2.04	0.945	0.87	0.45, 1.68	0.689	1.37	0.47, 3.95	0.561	2.22	0.70, 6.97	0.171
Homeless	1.41	0.79, 2.51	0.243	1.68	0.98, 2.86	0.056	2.05	0.94, 4.44	0.067	2.34	0.89, 6.09	0.081
Age at first sex, y (≤ 18 vs. > 18)	0.47	0.29, 0.77	0.003	0.45	0.26, 0.75	0.002	0.71	0.29, 1.71	0.450			
Age at sex work debut (≤ 18 vs. > 18)				1.48	0.85, 2.56	0.166	1.29	0.57, 2.90	0.533			
Duration of sex work (< 5 vs. ≥ 5)	1.25	0.69–2.25	0.453	1.36	0.85, 2.56	0.362	3.49	0.91, 13.27	0.067			
Number of clients per month												
≤ 5	Ref			Ref			Ref			Ref		
6–15	1.23	0.69, 2.20	0.474	1.96	1.11, 3.42	0.019	1.48	0.62, 3.50	0.369	2.55	0.93, 6.98	0.068
16–30	1.98	0.99, 3.96	0.052	3.12	1.64, 5.92	< 0.001	1.16	0.45, 2.99	0.744	1.88	0.60, 5.91	0.275
> 30	2.76	1.29, 5.90	0.009	5.76	2.82, 11.72	< 0.001	0.58	0.19, 1.70	0.324	0.23	0.02, 2.00	0.185
Group sex (ever) (Yes vs. No)							2.44	1.21, 4.92	0.013			
Ever consumed alcohol (Yes vs. No)	3.03	1.92, 6.79	< 0.001	1.90	1.22, 2.97	0.005	2.74	1.20, 6.20	0.016	1.88	0.78, 4.50	0.157
Ever incarcerated (Yes vs. No)	7.65	3.89, 15.30	< 0.001	3.05	1.85, 5.01	< 0.001	5.06	2.48, 10.28	< 0.001			

^a^Using multivariable logistic regression, variables with a *P* value less than 0.2 in the bivariable analysis were entered into multivariable analysis

### Injection drug use and associations with covariates

Lifetime injection drug use was higher among FSWs who reported primary school education (35.9%), earned $230 to $1100 (51.6%), were divorced (35.2%), reported living with family, friends, partner or spouse (45.9%), ever consumed alcohol (80.7%), and had ever incarcerated (61.5%). Injection drug use in the past month was significantly higher among participants who reported had not a history of incarceration (61.7%) (Table 1).

The multivariable logistic regression model showed that lifetime injection drug use was more likely to be reported by FSWs who ever practiced group sex (AOR 2.44, 95% CI 1.21, 4.92), ever consumed alcohol (AOR 2.74, 95% CI 1.20, 6.20), ever incarcerated (AOR 5.06, 95% CI 2.48, 10.28), and lifetime drug injection was less likely to be reported by FSWs who were married (AOR 0.11, 95% CI 0.01, 0.57), earned $2200 or more per month (AOR 0.22, 95% CI 0.05, 0.91) (Table 3).

## Discussion

The findings showed that about half of the street-based FSWs in Iran reported non-injection drug use in the past month, and almost one in ten had ever injected drugs, and 3.7% injected drugs in the past month. Non-injection and injection drug use was significantly associated with being divorced and temporary marriage, lower income, a higher number of clients, alcohol use, and incarceration. Moreover, injection drug use was associated with group sex practice.

Our results are comparable with global literature and recent literature from Iran on the prevalence of drug use among FSWs. The prevalence of drug use in this study falls within the range of international studies, which have reported drug use prevalence ranging from 2.6% recent use in China [26] to over 90% lifetime use in Australia [13]. Drug use prevalence that we found among street-based FSWs were higher than that reported for facility-based FSWs in previous studies in Iran. For example, Shokoohi et al. [20] using national bio-behavioral surveillance survey data, showed that only 24.9% of facility-based FSWs reported past-month non-injection drug use. Moreover, lifetime injection drug use prevalence was reported as 6.1% in 2015 and 14.6% in 2010 national bio-behavioral surveillance surveys with facility-based participants [18, 21]. The high prevalence of lifetime drug use among FSWs also reported among FSWs in Mazandaran (59.0%) [27] and shiraz (69.9%) [28] in the previous studies with small sample size. Accordingly, Harm reduction programs should prioritize drug use among FSWs to improve the impact of harm reduction among these marginalized population, particularly among those who street-based.

We found that socioeconomic factors including lower income, being divorced and temporary marriage associated with increased odds of using non-injection and injection drug use among our participants. The finding was consistent with prior studies in Iran that illustrated the role of lower socioeconomic status on FSW’s drug use engagement [29]. Another study in our context also showed the link between drug use and temporary marriage; FSWs who engaged in temporary marriage are more likely to be involved in drug use. Reasons offered from this study include temporary marriage can be a mediator for lack of familial support, low socioeconomic status, and sex work of the study participants, which is associated with an increased risk of drug use [20]. It is also documented that low socioeconomic status often heightens women’s entry into sex work and increases sex work related adverse health consequences including drug use [30]. For example, a study in the Dominican Republic revealed that employment stability reduced the likelihood of drug use by 60% in their participants [2]. We also suggest that economic promotion can decrease sex work-related adverse health consequences.

Our findings indicate the role of interpersonal and individual factors in elevating the odds of non-injection and injection drug use among street-based FSWs. We observed that higher number of clients, alcohol use, and group sex was associated with higher likelihood of non-injection and injection drug use among FSWs. FSWs who have higher number of clients may use or inject drugs to facilitate the process of engaging sexually with higher number of clients [3] and coping with their working circumstances [31]. A study on crystal methamphetamine use of FSWs in Iran also reported the association of drug use and having more sexual partners [22]. Group sex, an indicator of high-risk behaviors, has also been reported to be associated with injection and non-injection drug use [32, 33]. Our findings demonstrate the need to highlight the significance of drug treatment interventions for FSWs, particularly among those who report higher number of clients and who practicing group sex.

We also documented that a structural factor such as history of incarceration increase the likelihood of non-injection and injection drug use among FSWs. This finding are also consistent with previous studies indicating the role of this structural determinant on increased risk of non-injection and injection drug use among FSWs [20, 34]. Therefore, given the vulnerabilities associated with non-injection and injection drug use among FSWs and the fact that FSWs with a history of incarceration were more likely to used or inject drugs, our findings, in line with the evidence among FSWs in Iran, suggested a need for removing the barriers to seeking drug use treatment, targeting the structural determinants of drug use among FSWs, and providing effective drug use prevention and treatment programs for FSWs to address and reduce associated harms and vulnerabilities among FSWs [20, 34].

Our study had three significant limitations. First, we measured the drug use and drug injection by self-report; thus, the obtained data might be under-reported due to social desirability bias. Secondly, like other cross-sectional studies, we could only assess the factors correlated with drug use and drug injection, but not the cause and effect relationships. Finally, to recruit street-based FSWs from venues, we used a non-probability sampling technique, which limited the generalizability of our findings.

## Conclusion

Regardless of these limitations, the present study contributes to the growing body of evidence on drug use among FSWs in Iran. Our research showed that non-injection drug use is prevalent among street-based FSWs, and the prevalence of injection drug use among this sample of FSWs is concerning. Findings suggest the need to harm reduction interventions such as behavioral and opioid substitution therapeutic strategies that focus on street-based FSWs in Iran to reduce harm among this understudied and marginalized population. Future studies addressing access of street-based FSWs to harm reduction services are recommended.

## Data Availability

The datasets used and/or analyzed during the current study are available from the corresponding author on reasonable request.

## Abbreviations

OR: Odds ratio
CI: Confidence intervals
FSWs: Female sex workers
